# Coronary heart disease evaluation using PCAT radiomics model based on coronary CT angiography and pericoronary adipose tissue

**DOI:** 10.1097/MD.0000000000039936

**Published:** 2024-10-18

**Authors:** Chuanmin Zhang

**Affiliations:** a Department of Radiology, Taikang Xinlin Drum Tower Hospital, Affiliated Hospital of Medical School, Nanjing University, Nanjing, China.

**Keywords:** coronary arteries, coronary heart disease, CT angiography, lesion samples, radiomics features, surrounding adipose tissue

## Abstract

To explore the clinical application value of radiomics model based on pericoronary adipose tissue (PCAT) in predicting coronary heart disease. A retrospective analysis was performed for inpatients who had undergone coronary computed tomography angiography from January to December 2023, and 164 cases of coronary artery lesions were screened as the lesion group and 190 cases of normal coronary artery samples were selected as the control group. The clinical data and imaging data of all patients were collected, the radiomics features were extracted by relevant software, and the “region of interest” of pericoronary fat was delineated, and the selection operator and multivariate logistic regression were used to screen the radiomic features of pericoronary fat. A coronary heart disease evaluation model was constructed by the best radiomics features. Area under the curve values of the PCAT radiomics scoring model for predicting the receiver operating characteristic curve of coronary heart disease were 0.863 and 0.851 in training and test sets, respectively. After calibration curve analysis, PCAT radiomics scoring model has a high consistency between the predictive evaluation results and the actual results of coronary heart disease events. In addition, in the training set, the PCAT radiomics scoring model has a net benefit on all threshold probabilities. In the test set, the model has a negative net return with only a small number of threshold probabilities. After combining the clinical characteristics model, the evaluation accuracy of the model for coronary heart disease can reach 0.896. PCAT radiomics model based on coronary computed tomography angiography can effectively predict and evaluate coronary heart disease, which is of great value for the clinical diagnosis of coronary artery disease.

## 1. Introduction

Coronary heart disease is a common cardiovascular disease with a high mortality rate, and early and accurate assessment and prediction of coronary heart disease is essential for subsequent diagnosis and treatment.^[1,2]^ Traditional methods for assessing the disease include clinical symptoms, electrocardiograms, and blood biochemistry, but these methods have certain limitations.^[3]^ The development of coronary computed tomography (CT) angiography technology has provided a new noninvasive evaluation of coronary artery lesions. This angiography is a noninvasive procedure that provides high-resolution images of the coronary arteries to help clinicians accurately assess the anatomy and lesions of the coronary arteries.^[4,5]^ However, relying solely on the manual judgment of physicians to assess coronary artery disease carries a certain degree of subjectivity and risk of misjudgment.^[6]^ The latest studies have shown that pericoronary fat radiomics combined with CT angiography images and perfusion imaging of coronary arteries can more accurately assess the degree of coronary artery lesions and blood perfusion through feature extraction and analysis of image data. Therefore, in order to evaluate coronary artery lesions more accurately, the feature selection method was introduced into the processing of imaging data of coronary CT angiography, and these feature data constructed a prediction model to evaluate coronary artery lesions severity and predict coronary heart disease.

## 2. Information and methodology

### 2.1. General information

The data of patients in Taikang Xianlin Drum Tower Hospital from January to December 2023 were retrospectively analyzed. Three hundred fifty-four patients with coronary CT angiography for suspected cardiovascular disease were selected as the experimental subjects, including 221 male patients and 133 female patients, aged 30 to 89 years.

The inclusion criteria were: 1. The clinical diagnosis data of the patients were complete. 2. Coronary CT angiography images are clear. 3. The patient underwent DSA examination at the same time before surgery. 4. All patients take the same type of coronary heart disease drugs for treatment. 5. The patients had plaques and stenosis, and were divided into mild stenosis group (stenosis rate ≤ 50%), moderate stenosis group (50% ≤ stenosis rate ≤ 70%) and severe stenosis group (70% ≤ stenosis rate) according to the stenosis rate.

Exclusion criteria: 1. The patient has other conditions such as a history of congenital heart disease or heart failure and cannot cooperate with the completion of the experiment. 2. The patient has undergone other coronary artery surgery before surgery. 3. The image artifact of CT angiography examination of the patient is serious.

The entire experiment has been approved by our Ethics Committee, and the informed consent form has been signed by patients and their families.

### 2.2. Research methodology

#### 2.2.1. CT scan method

The 256-slice 512-slice Revolution CT produced by GE in the United States was used to detect the coronary arteries of the patient’s heart. The scanning range was 2 cm from the tracheal tumina to the diaphragm. The scanning parameters of the instrument were set to 100 kV, the reconstruction thickness was set to 0.625 mm, the layer spacing was set to 0.625 mm, and the tube current was set to Smart mA (300–545 mA). The contrast agent iohexol (iodine content 350 mgL/mL, Beijing Beilu Pharmaceutical Co., Ltd., Beijing, China) was injected through the vein of the right elbow at a flow rate of 5 mL/s, and then 45 mL of normal saline was injected. When the CT value in the set area of interest reached or exceeded the threshold, the coronary artery scan was started with a delay of 10 seconds, and the image was uploaded to the GEAW472 station after the scanning was completed.

#### 2.2.2. CT-FFR measurement method

The deep pulse fraction DVFFR software was used for noninvasive CT-FFR detection, which can automatically and quickly calculate the FFR value at any point in the coronary artery tree using an intelligent algorithm.^[7]^ A copy of the coronary CT angiography sequence images of at least 1 patient who was admitted to our hospital for coronary CT angiography from January to December 2023 and had a coronary stenosis rate of more than 50% was copied and transmitted to the deep pulse fraction DVFFR software for analysis, and then all CT-FFR reports of the study subjects were exported, and the reports were statistically analyzed according to the standards.

#### 2.2.3. PCAT region of interest delineation

**Figure 1.**
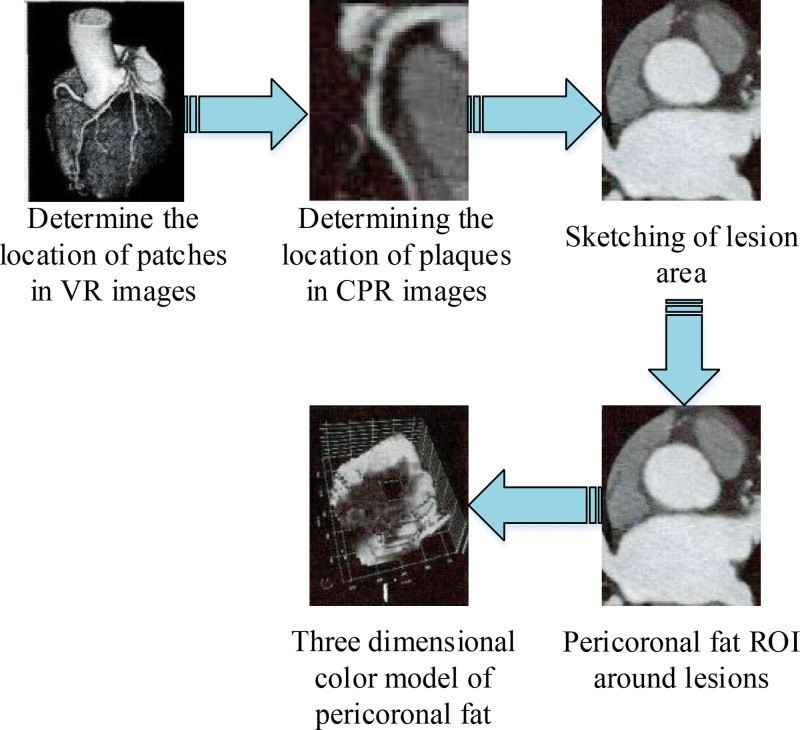
Flowchart of plaque delineation.

The coronary images of patients in the specific lesion group and the corresponding control group were screened out in the CT-FFR analysis report of the patients. Two radiologists were selected to observe the location and length of the plaque at its strictest part on the lesion coronary artery reconstruction image, and then the region of interest was delineated layer by layer on the coronary artery where the plaque is located using the ITK-SNAP software.^[8]^ When analyzed, the extent of the specific lesion determines the longitudinal length of the pericoronary adipose tissue (PCAT). According to the study, 98.3% of the most stenotic coronary arteries in the specific lesion group were located in the left anterior descending artery and the right coronary artery. Therefore, the location and length of the plaques were counted, and the standard deviation and mean were calculated, respectively, and the position and length of the normal group were used as the benchmark. After the delineation is completed, the “Fat Segmentation” function of the ITK-SNAP software can automatically generate a single-layer image and a three-dimensional stereoscopic image of the area of interest of the pericoronal fat based on the delineated plaques (the CT value of pericoronal fat was set to ‐25 to 175 HU).^[9]^ The plaque delineation procedure is shown in Figure 1.

After the images of the pericoronal fat region of interest were automatically generated by the ITK-SNAP 4.0 software, 1765 radiomics features of each pericoronal fat region of interest were extracted by the “Radiomics Calculate” function of the software.

#### 2.2.4. Radiomics feature data preprocessing and model construction

For the collected radiomics features, the univariate correlation analysis method was used to screen the features, then the selection operator was used for re, and then the multivariate logistic regression method was used for step-by-step selection. For the collected coronary art dataset, it was divided according to the ratio of test set: dataset = 3:7. The training set screened the optimal features, and the screened subsets were used to construct the radiomics model.

### 2.3. Statistical analysis

R3.5.1 and Python 3.5.6 were used to analyze all statistical results.^[10]^ $\bar{x}\pm s$ presented measurement data, and one-way ANOVA compared between the 3 groups.^[11]^ The frequency and rate described the metrics, the chi-square test analyzed the data, and finally the receiver operating characteristic (ROC) curve was used for model evaluation and analysis.

## 3. Results

### 3.1. Characteristics of the patient’s clinical case

In this study, 164 coronary samples were collected from lesion group and 190 patients from control group. Their case characteristics are shown in Table 1.

**Table 1:** Baseline data comparison between 2 groups.

Features	Unit	Single factor logistic regression		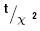	*P*-value	Single factor logistic regression
		Lesion group (164)	Control group (190)			*P*-value
Man	Person	102	123	3.965*	.014	NA
Woman	Person	62	77			
Age	Year	60.35 ± 10.32	61.11 ± 19.93	0.119†	.012	NA
History of smoking	Year	74	34	0.235*	±.001	.002
Hypertension	Example	65	32	0.496*	≤.001	≤.001
Hyperglycemia	Example	71	38	0.045*	.021	NA
Hyperlipidemia	Example	62	21	2.365*	.065	NA
Triglycerides	mmol/L	4.23 ± 1.32	40.6 ± 1.10	1.124*	.549	NA
High-density lipoprotein	mmol/L	1.71 ± 0.94	1.68 ± 0.85	1.235*	≤.001	.002
Low-density lipoprotein	mmol/L	2.42 ± 0.63	2.36 ± 0.90	1.203*	.442	NA
Stenosis rate	%	68.36 ± 20.31	50.36 ± 20.31	2.012†	≤.001	≤.001
DO	HU	‐67.74 ± 10.1	‐67.65 ± 9.1	1.635†	.624	NA

*Note*: In the table, FAI is the fat attenuation index.

* Is the value χ2.

† Is the value *t*.

As can be seen from Table 1, except for the *P* value of “hyperlipidemia,” “triglycerides,” and “FAI,” which was >0.5, the differences in the other indicators were statistically significant.

### 3.2. Consistency analysis

**Figure 2.**
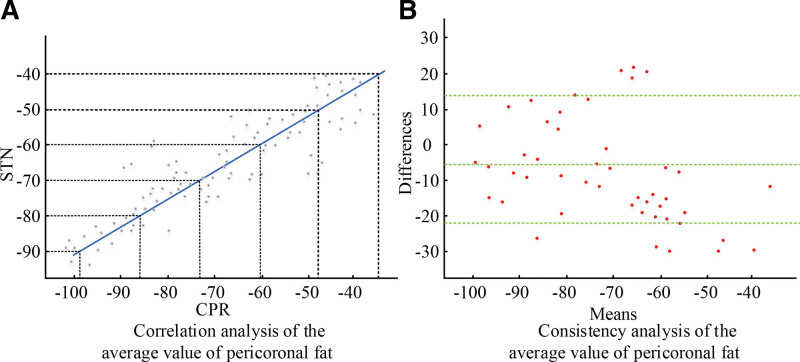
Correlation and consistency analysis of the mean value of pericrown fat.

The method in Section 1.2.3 was used to delineate the “region of interest” of pericoronary fat between the patient’s ordinary cardiopulmonary resuscitation (CPR) images and the straightened CPR images, and then the correlation and consistency between the two were analyzed in Figure 2.

**Figure 3.**
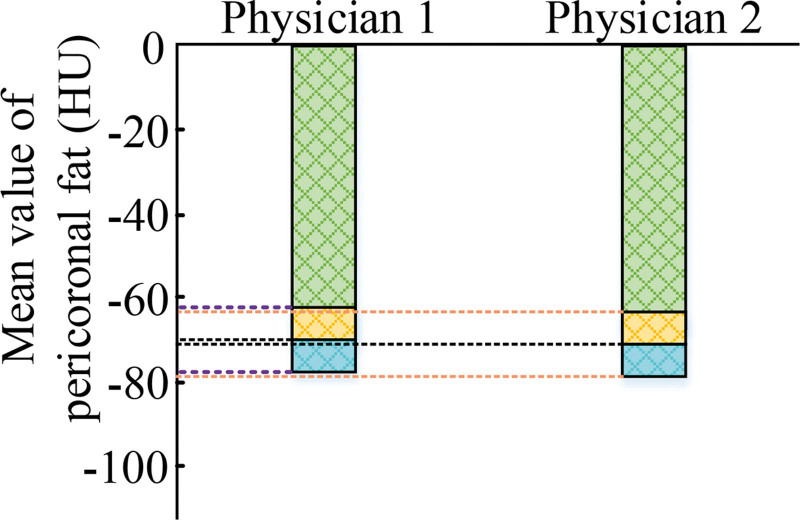
Average pericrown fat measurements by 2 radiologists.

In Figure 2, the correlation coefficient of the mean value of pericoronal fat was about 0.8 (*P* < .001), the 95% confidence interval of the difference between the two was (‐23.36 to 14.65), and the mean difference was 1.63 HU. The average CT values of pericoronal fat plotted by 2 radiologists in the experiment are shown in Figure 3.

As can be seen from Figure 3, the measurement result of radiologist 1 was ‐71.36 ± 16.32 HU, and the measurement result of radiologist 1 was −72.12 ± 15.96 HU. Therefore, it can be seen that the intra-correlation coefficient of the mean pericoronal fat measured by the 2 radiologists was 0.963. Since the correlation coefficient within this group was >0.80, it indicated that there was a high degree of agreement between the measurement results of the 2 doctors, and it also verified the high reliability of the method used in the study.

### 3.3. Construction of PCAT radiomics model and effect analysis on the evaluation of coronary heart disease

#### 3.3.1. Radiomics model construction

**Figure 4.**
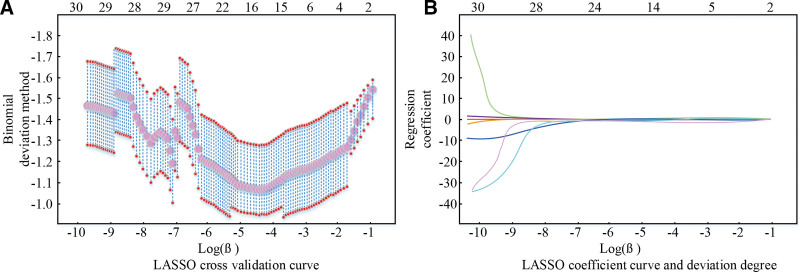
LASSO software performs the feature screening process. LASSO = least absolute shrinkage and selection operator.

After the collected radiomics features were preprocessed, and the redundant and unnecessary features were removed, there were 28 radiomics features left, and the least absolute shrinkage and selection operator (LASSO) algorithm screened out the optimal subset of features to construct an electromics model to evaluate the coronary heart disease of patients. The process of feature screening using the LASSO algorithm is shown in Figure 4.

**Figure 5.**
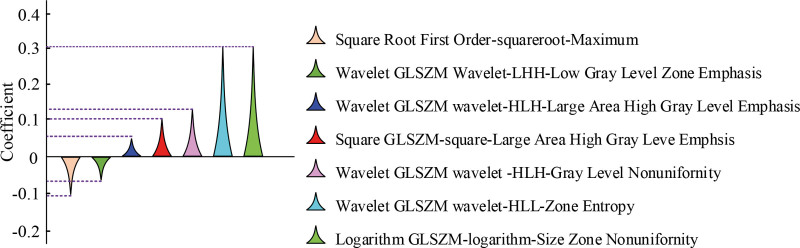
Contribution of regression coefficients in the construction of feature models.

Figure 4A shows the LASSO cross-validation curves, and Figure 4B shows the results of describing the LASSO coefficient curves versus deviation in the constructed impactomics features. Entering the optimal coefficient *β* = 0.030 during the screening process resulted in 7 nonzero coefficients, which led to the identification of the imageomics features for subsequent experiments. The 7 nonzero coefficients included Square Root First Order-Squareroot-Maximum, Wavelet GLSZM Wavelet-LHH-Low Gray Level Zone Emphasis, Wavelet GLSZM Wavelet-HLH-Large Area High Gray Level Emphasis, Square GLSZM-Square-Large Area High Gray Level Emphasis, Wavelet GLSZM Wavelet-HLH-Gray Level Nonuniformity, Wavelet GLSZM Wavelet-HLL-Zone Entropy, and Logarithm GLSZM-Logarithm-Size Zone Nonuniformity. The selected coefficients in the contribution to the construction of the imaging histology features is shown in Figure 5.

**Figure 6.**
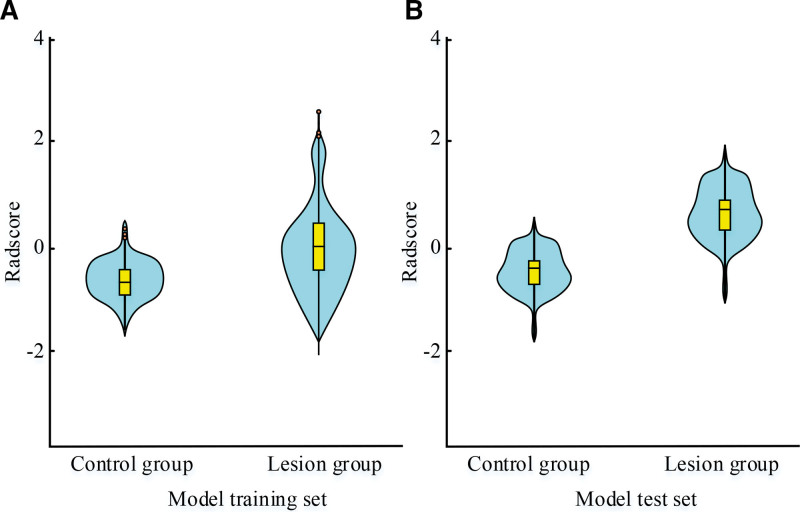
Coronary artery stenosis severity scores of patients in 2 groups.

According to the contribution of each coefficient to the construction of the feature model, the corresponding coefficients of the 7 radiomics features were about −0.11, −0.07, 0.06, 0.10, 0.12, 0.30, and 0.30, respectively, and the evaluation model of coronary heart disease was constructed according to these coefficients, and the results of coronary stenosis severity scores are shown in Figure 6.

Figure 6A shows the experimental results of the training set, which showed that the severity of coronary stenosis was quite different, and the Radscore score was higher in the patients in the coronary lesion group. Figure 6B shows the experimental results in the test set, which showed that there was little difference in the severity of coronary artery stenosis, but the Radscore score was still higher in the coronary lesion group. This indicated that in the case of *P* < .05, a significant difference showed in the Radscore score between 2 groups, which could evaluate the coronary artery lesions of the patients.

#### 3.3.2. Validation analysis of PCAT radiomics model evaluation

**Figure 7.**
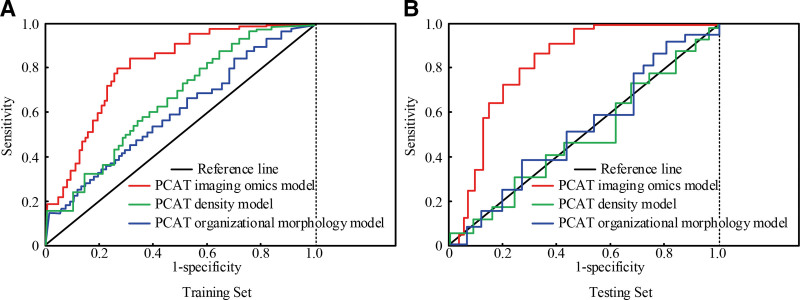
ROC curves of the 3 PCAT models in the training set and the test set. PCAT = pericoronary adipose tissue, ROC = receiver operating characteristic.

According to the LASSO coefficient analysis, the PCAT radiomics scoring model was constructed, the PCAT density model was constructed using average CT density value of the extracted PCAT radiomics features, and the PCAT tissue morphology model was constructed based on the extracted PCAT histomorphological features. The ROC curves of the 3 models for predicting coronary heart disease were plotted, and the results are shown in Figure 7.

**Figure 8.**
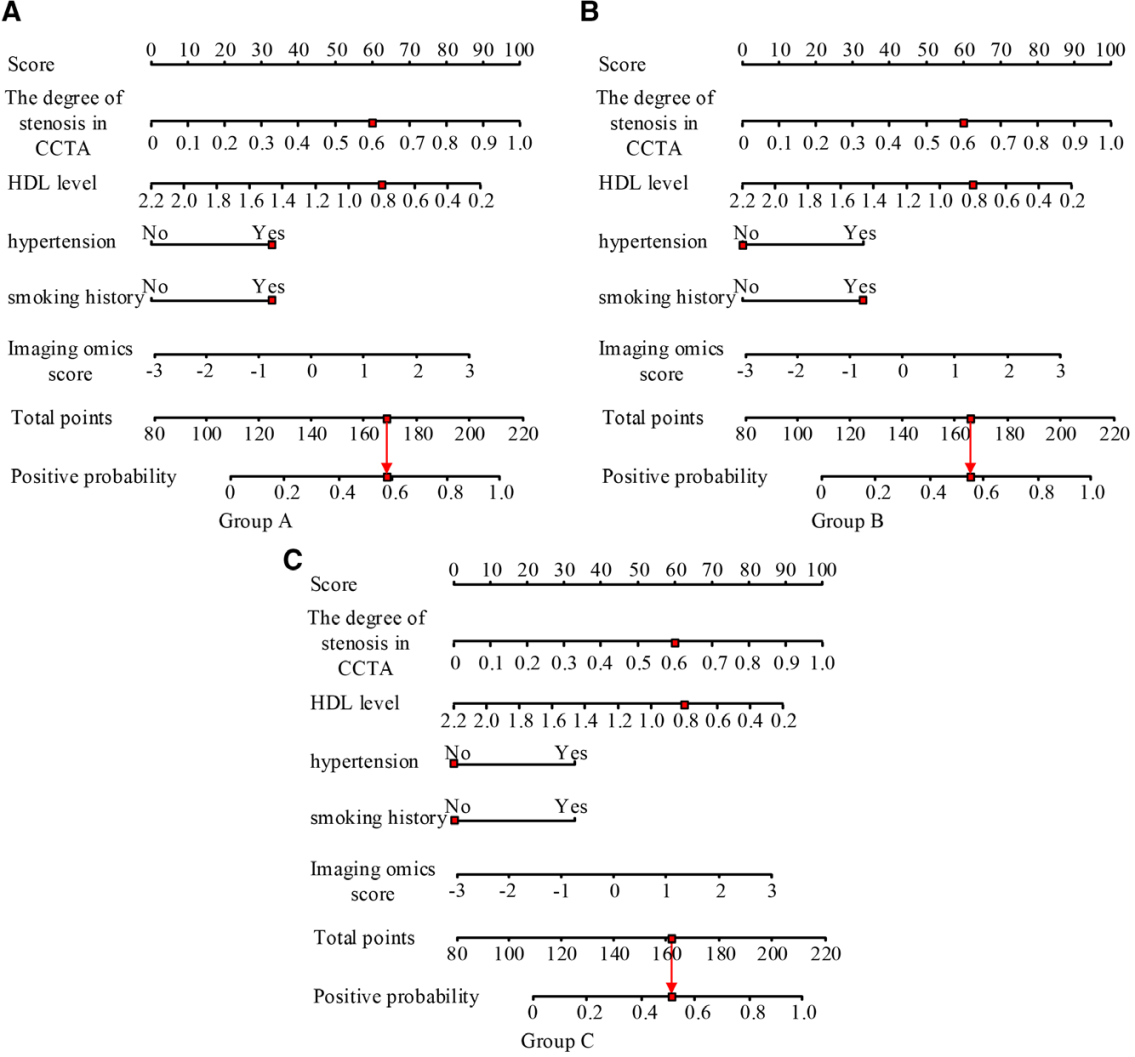
Nomogram of coronary lesion assessment of patients.

In Figure 7A, the area under the curve (AUC) values of the PCAT radiomics scoring model, PCAT density model, and PCAT tissue morphology model were 0.863, 0.693, and 0.634 in the training set (*P* < .05). The results showed that PCAT radiomics scoring model used in the study was better than the other 2 models in the evaluation of coronary heart disease. In Figure 7B, AUC of PCAT radiomics scoring model, PCAT density model and PCAT tissue morphology model were 0.851, 0.563, and 0.523 in the training set (*P* = .05). AUC of the PCAT radiomics scoring model was still the highest, which further verified the evaluation effect of the model. On the whole, the results of the testing set were consistent with the training set, and AUC of ROC curve of the PCAT radiomics scoring model was the largest. To further verify the evaluation ability of the PCAT radiomics scoring model, a nomogram model was constructed based on the training set, combined with the degree of coronary artery stenosis, high-density lipoprotein level, Radscore score, history of hypertension, and history of smoking. The results are shown in Figure 8, and the red color in Figure 8 indicates the case.

**Figure 9.**
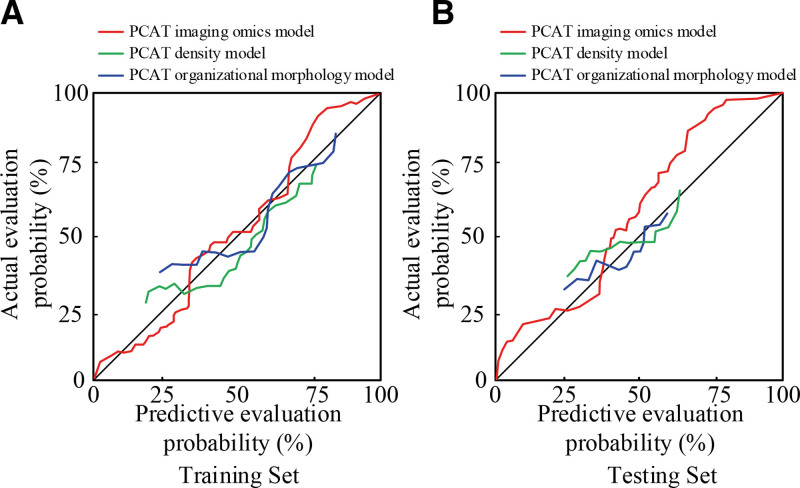
Calibration curves of the 3 PCAT models in the training set and the test set. PCAT = pericoronary adipose tissue.

As can be seen from Figure 8C, the risk probability of coronary artery disease was accurately assessed by combining the patient’s coronary artery stenosis, high-density lipoprotein level, Radscore score, and other parameters. As can be seen from Figure 8A, if a patient had hypertension and smoking, the disease probability in the case shown in the figure was 0.59. As can be seen from Figure 8B, if a patient had a history of smoking, the probability of coronary artery disease in the case shown in the figure was 0.57. As can be seen from Figure 8A, if the patient did not have a history of hypertension and smoking, the probability of coronary artery disease in the situation shown in the figure was 0.52. This suggested that the patient’s coronary artery lesions were also affected by other factors. Calibration curve analysis verified nomogram fit in the application of the model to assess coronary lesions, and the calibration curves of the 3 models are shown in Figure 9.

**Figure 10.**
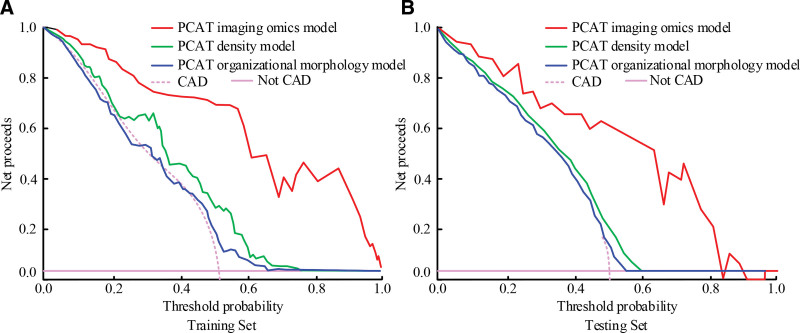
Decision curves of the 3 PCAT models in the training set and the test set. PCAT = pericoronary adipose tissue.

In Figure 9A, in the training set, the agreement between the predictive assessment results and the actual results for the patients’ coronary lesion events was obtained for the PCAT imaging histology scoring model (*P* = .365) > PCAT density model (*P* = .468) > PCAT histomorphometry model (*P* = .469). In Figure 9B, the same results were obtained in the test set. This indicates that the calibration curves for assessing the column-line diagrams of coronary lesions showed the highest agreement between the estimated and predicted values of the PCAT imaging histomorphometric scoring model constructed in the study. Clinical decision curves were used to analyze the clinical value of coronary lesion status prediction, and the clinical decision curves are shown in Figure 10. The clinical diagnostic intervention was defined as routine diagnosis for all patients, and no diagnosis was defined as no diagnosis for anyone who did not intervene. The results of evaluating the clinical value of predictions assessed by the PCAT imaging histology scoring model, the PCAT density model, and the PCAT histomorphometry model in patients with coronary artery disease are shown in Figure 10.

In Figure 10A, in the training set, the PCAT radiomics scoring model had a net benefit in the whole threshold probability, while the PCAT density model had a net benefit of 0.76 when the threshold probability reached 0.76, and the PCAT tissue morphology model had a net benefit of 0.70 when the threshold probability reached 0.70. The results indicated that the clinical application value of the PCAT radiomics scoring model constructed was significantly higher than that of the 2 models. In Figure 10B, in the test set, the PCAT radiomics scoring model had a negative net return only with a small number of threshold probabilities. The PCAT density model had a net return of 0.55 when the threshold probability reached 0.55, and the CAT tissue morphology model had a net return of 0 when the threshold probability reached 0.59. The clinical application value of the PCAT radiomics scoring model was further verified.

#### 3.3.3. Effectiveness of clinical features combined with PCAT imaging histology modeling assessment

The above experiments confirmed that the PCAT radiomics scoring model constructed by the study accurately evaluated and predicted the coronary lesions experienced by patients. However, the accuracy of the assessment was still further improved, and the clinical characteristics model adopted by Wu et al had a good effect on the evaluation of medical diseases through literature review.^[12]^ Therefore, this study fused the clinical feature model to construct a joint model based on the PCAT radiomics scoring model to improve the evaluation accuracy of the original model. The same dataset was used to test the performance of these 3 models in assessing coronary heart disease in Table 2.

**Table 2:** Test results of the performance of the 3 models for coronary heart disease assessment.

	PCAT radiomics scoring model	Clinical characteristic model	Joint model
	Training set		
Precision	0.836 [0.816–0.840]	0.812 [0.811–0.820]	0.896 [0.890–0.910]
AUC value	0.863 [0.856–0.870]	0.853 [0.850–0.860]	0.883 [0.880–0.890]
Sensitivity	0.63	0.69	0.79
Specificity	0.84	0.81	0.78
Positive predictive value of coronary heart disease	0.79	0.80	0.85
Negative predictive value of coronary heart disease	0.79	0.75	0.88

	Test set		
Precision	0.796 [0.790–0.810]	0.816 [0.810–0.30]	0.889 [0.880–0.910]
AUC value	0.851 [0.850–0.860]	0.835 [0.830–0.840]	0.896 [0.890–0.910]
Sensitivity	0.76	0.71	0.82
Specificity	0.83	0.85	0.88
Positive predictive value of coronary heart disease	0.76	0.71	0.84
Negative predictive value of coronary heart disease	0.73	0.79	0.72

*Note*: The accuracy and AUC values in the table are within 95% confidence intervals.

AUC = area under the curve, PCAT = pericoronary adipose tissue.

In Table 2, the accuracy and AUC of the PCAT radiomics scoring model in the training set were 0.836 and 0.863, while the accuracy and AUC of the combined model in training set were 0.896 and 0.883. In contrast, this combined model was verified to have better performance in assessing coronary heart disease than the PCAT radiomics scoring model. In test set, the accuracy and AUC values of PCAT radiomics scoring model were 0.796 and 0.851, respectively, while the accuracy and AUC values of the combined model were 0.889 and 0.896, respectively. It further demonstrated that the joint model had better performance when assessing coronary heart disease than the PCAT radiomics scoring model. From the perspective of the indicators of the whole table, the accuracy, AUC value, sensitivity, and specificity of the joint model to evaluate coronary heart disease were better in both sets than those of the CAT radiomics scoring model and the clinical characteristics model alone.

## 4. Discussion

Coronary heart disease is caused by insufficient blood supply to the coronary arteries, which are the main blood vessels that supply blood to the heart, and they are responsible for transporting oxygen- and nutrient-rich blood to the heart muscle. PCAT is in direct contact with the coronary artery wall, and patients with coronary lesions often have vascular inflammation, which can affect the patient’s pericoronary fat through paracrine, thereby affecting the CT value of pericoronary fat on coronary CT angiography.^[13]^ Studies have shown that the CT value of peripheral adipose tissue in patients with coronary lesions is different from that of normal people. At present, CT angiography technology is becoming more and more mature and has become an important means for clinical evaluation of coronary lesions in hospitals. Tricarico et al have demonstrated that CT angiography is highly accurate in assessing the degree of coronary artery stenosis.^[14]^ The results of the study showed that CT angiography accurately assessed 36 cases of mild stenosis, 31 cases of moderate stenosis, and 29 cases of severe stenosis in patients with coronary lesions, which was consistent with the results of digital subtraction angiography, the “gold standard” of coronary stenosis, with a rate of 92.3%. Coronary CT angiography can clearly observe the relationship between coronary artery vascular lesions and surrounding adipose tissue by injecting iohexol contrast agent into the patient’s blood vessels and scanning the coronary arteries with multidetector spiral CT, which can be analyzed according to the actual situation presented by the images, so as to improve the accuracy of coronary CT angiography in assessing stenosis degree.^[15,16]^

Coronary artery disease usually refers to the formation of plaque in blood vessels walls in the coronary arteries, which can block blood flow and affect the blood supply to the heart. Coronary CT angiography can be used to evaluate and predict this condition, but the accuracy of this test is affected by the surgical radiologist’s experience in post-processing and diagnostic diagnosis.^[17]^ The references showed that this detection method had nonness, high efficiency, convenience and rapidity, and low cost, and has long become a common method to evaluate coronary heart disease.^[18]^ Coronary CT angiography can evaluate coronary artery stenosis and analyze the adipose tissue around the coronary arteries to determine the condition of coronary artery lesions. Because the reconstruction method is affected by the delineation of the “region of interest,” the study performed pericoronary fat delineation and analysis of the patient’s ordinary CPR images and straightened CPR images, and the correlation coefficient between the two was about 0.8, indicating that there was a high correlation and consistency between the two, which proved the reproducibility of the research method. At the same time, the intragroup correlation coefficient of the average pericoronal fat measured by 2 radiologists in the experiment was 0.963. It showed a high degree of consistency between the measurement results of the 2 doctors, which verified the reliability of the method.

The references showed that the average CT value of pericoronal fat helped identify vulnerable plaques, which was related to the probability of coronary heart disease, left ventricular hypertrophy, in vivo inflammation level, and myocardial perfusion downstream of in vivo plaques, indicating that the average CT value of pericoronal fat was a valuable radiomics marker. Results showed that the CT value of the coronary artery lesion group was −71.52 ± 17.36 HU, while the CT value of the control group was −70.41 ± 16.89 HU (*P* < .05). At the *s* < e time, some data showed that multiparameter based radiomics models had better evaluation performance than single-parameter models. Therefore, the construction of the follow-up evaluation model extracted multiple parameters from the pericrown fat and screened them.

At present, most coronary CT angiography radiomics studies focus on coronary plaques, and studies have innovatively analyzed PCAT. According to the data, the appearance of the adipose tissue around the coronary arteries is mainly gray and black, which is difficult to distinguish with the naked eye. Radiomics is a method that uses medical imaging data for quantitative analysis and pattern recognition. By extracting and analyzing features in medical images, the properties of tissues and lesions are represented. Coronary artery disease is closely related to the surrounding adipose tissue, so the radiomics model of PCAT can be constructed based on coronary CT angiography for the evaluation of coronary heart disease. In this study, 1765 radiomics features were extracted, 7 representative best features were screened by LASSO regression and multivariate logistic regression, and a PCAT radiomics scoring model was constructed using a subset of optimal features.^[19,20]^ According to the results, the 7 features screened out were all information features that reflected PCAT, and the coronary artery morphology between the coronary artery lesion group and the control group would not have an impact on the construction of the radiomics model. To verify model evaluation effect on coronary heart disease, the PCAT density model, PCAT tissue morphology model and comparative experiments were conducted. The results showed that AUC of the PCAT radiomics scoring model, PCAT density model and PCAT tissue morphology model were 0.863, 0.693, and 0.634 in training set, respectively. The results showed that the PCAT radiomics scoring model effectively evaluated coronary heart disease. After the calibration curve analysis, the PCAT radiomics scoring model > PCAT density model > PCAT tissue morphology model was used to determine the consistency between the predictive evaluation results and the actual results of coronary lesion events. The evaluation effect of PCAT radiomics scoring model is further illustrated. In addition, in the decision curve, the PCAT radiomics scoring model showed high clinical significance.

Coronary heart disease has become a common disease, and traditional coronary CT angiography can provide information about the anatomical structure of the patient’s coronary arteries, but it cannot reflect physiological information such as blood flow, blood supply, and myocardial function. Limitations of the study: The study design used a single-center retrospective study method and a small sample size, so there was a certain error in the experimental results. Subsequent experiments will include data from more other centers and expand the sample sources for in-depth research. In addition, there are still relatively few PCAT radiomics features extracted in the study, and follow-up studies will consider introducing modern heuristic algorithms to extract radiomics features more comprehensively and finely, so as to assist in early clinical identification of patients who may be at risk of coronary disease.

## 5. Conclusion

The PCAT radiomics scoring model constructed in this study can accurately predict and evaluate coronary heart disease. However, the model requires many data to train and validate. In training set, accuracy and AUC values of joint evaluation model were 0.896 and 0.883, sensitivity and specificity were 0.79 and 0.78, and positive and negative predictive values for coronary heart disease were 0.85 and 0.88, respectively, which greatly improved the evaluation accuracy of the original model for coronary heart disease.

## Author contributions

**Conceptualization:** Chuanmin Zhang.

**Data curation:** Chuanmin Zhang.

**Formal analysis:** Chuanmin Zhang.

**Investigation:** Chuanmin Zhang.

**Methodology:** Chuanmin Zhang.

**Writing – original draft:** Chuanmin Zhang.

**Writing – review & editing:** Chuanmin Zhang.

## References

[1] Goldbourt U Grossman E. Blood pressure variability at midlife is associated with all-cause, coronary heart disease and stroke long term mortality. J Hypertens. 2020;38:1722–8. 32371770 10.1097/HJH.0000000000002447

[2] Tran B. Assessment and management of peripheral arterial disease: what every cardiologist should know. Heart. 2021;107:1825–43. 10.1136/heartjnl-2019-316164 PMC8562307 33985986

[3] Davidson LJ Davidson CJ. Transcatheter treatment of valvular heart disease: a review. JAMA. 2021;325:2480–94. 34156404 10.1001/jama.2021.2133

[4] Narvaez Linares NF Poitras M Burkauskas J. Neuropsychological sequelae of coronary heart disease in women: a systematic review. Neurosci Biobehav Rev. 2021;127:837–51. 34062209 10.1016/j.neubiorev.2021.05.026

[5] Cami E Tagami T Raff G Gallagher MJ Safian RD. Importance of measurement site on assessment of lesion-specific ischemia and diagnostic performance by coronary computed tomography angiography-derived fractional flow reserve. J Cardiovasc Comput Tomogr. 2021;15:114–20. 32943356 10.1016/j.jcct.2020.08.005

[6] Pu Z Hakim D Croce K Stone PH. Preemptive percutaneous coronary intervention for coronary artery disease: identification of the appropriate high-risk lesion. Curr Opin Cardiol. 2020;35:712–9. 32852346 10.1097/HCO.0000000000000789

[7] Ford TJ Ong P Sechtem U Beltrame J Berry C. Assessment of vascular dysfunction in patients without obstructive coronary artery disease. JACC Cardiovasc Interv. 2020;13:1847–64. 32819476 10.1016/j.jcin.2020.05.052 PMC7447977

[8] Heianza Y Ma W Didonato JA Sun Q Qi L. Long-term changes in gut microbial metabolite trimethylamine N-oxide and coronary heart disease risk. J Am Coll Cardiol. 2020;75:763–72. 32081286 10.1016/j.jacc.2019.11.060 PMC8140616

[9] Frommelt P Lopez L Vivian Dimas V. Recommendations for multimodality assessment of congenital coronary anomalies: a guide from the American society of echocardiography. J Am Soc Echocardiogr. 2020;33:259–94. 32143778 10.1016/j.echo.2019.10.011

[10] Mala ST Kiran BR. One-way ANOVA, Tukey HSD, Scheffe, Bonferroni and Holm multiple comparison tests for physico-chemical parameters in four water bodies of Davangere District, Karnataka. Int J Pharma Bio Sci. 2021;11:66–73.

[11] Tomá M Myllymki M Milan J Hahn U. A one-way ANOVA test for functional data with graphical interpretation. Cybernetics (Prague). 2020;56:432–58.

[12] Wu T Chen S Tian Y Wu P. A feature optimized deep learning model for clinical data mining. Chin J Electron. 2020;29:476–81.

[13] Zhong Z Zhang P Duan H Yang H Li Q He FA. Comparison between X-ray imaging and an innovative computer-aided design method based on weightbearing CT scan images for assessing hallux valgus—ScienceDirect. J Foot Ankle Surg. 2021;60:6–10. 32253154 10.1053/j.jfas.2018.12.044

[14] Tricarico R Chou TH Eisert SN Reinhardt J Stacy MR. Non-invasive molecular imaging of inflammation in tissue-engineered vascular grafts using 18F-FDG PET/CT. J Am Coll Cardiol. 2020;75:1768–9.

[15] Donato L Cecchi R Goldoni M Ubelaker DH. Photogrammetry vs CT scan: evaluation of accuracy of a low-cost three-dimensional acquisition method for forensic facial approximation. J Forensic Sci. 2020;65:1260–5. 32216148 10.1111/1556-4029.14319

[16] Hadavi N Nordin MJ Shojaeipour A Nasrudin MF. Classification of normal and abnormal lung CT-scan images using cellular learning automata. J Comput Sci. 2020;16:14–24.

[17] Siri S. A novel approach to extract exact liver image boundary from abdominal CT scan using neutrosophic set and fast marching method. J Intell Syst. 2020;28:517–32.

[18] Ravichandran S Nath N Jones DC Li G Mosca PJ. The utility of initial staging PET-CT as a baseline scan for surveillance imaging in stage II and III melanoma. Surg Oncol. 2020;35:533–9. 33161362 10.1016/j.suronc.2020.10.018

[19] Thaseen IS Kumar CA Ahmad A. Integrated intrusion detection model using chi-square feature selection and ensemble of classifiers. Arab J Sci Eng. 2019;44:3357–68.

[20] Talevi G Pannone L Monaco C. Evaluation of photogrammetry for medical application in cardiology. Front Bioeng Biotechnol. 2023;11:1044647. 36714012 10.3389/fbioe.2023.1044647 PMC9879954

